# Encouraging Expressions Affect the Brain and Alter Visual Attention

**DOI:** 10.1371/journal.pone.0005920

**Published:** 2009-06-17

**Authors:** Manuel Martín-Loeches, Alejandra Sel, Pilar Casado, Laura Jiménez, Luis Castellanos

**Affiliations:** 1 Center for Human Evolution and Behavior, UCM-ISCIII, Madrid, Spain; 2 Psychobiology Department, Complutense University of Madrid, Madrid, Spain; 3 Centro Estratégico de Investigación Neurocientífica “El Jardín de Junio”, Madrid, Spain; Tel Aviv University, Israel

## Abstract

**Background:**

Very often, encouraging or discouraging expressions are used in competitive contexts, such as sports practice, aiming at provoking an emotional reaction on the listener and, consequently, an effect on subsequent cognition and/or performance. However, the actual efficiency of these expressions has not been tested scientifically.

**Methodology/Principal Findings:**

To fill this gap, we studied the effects of encouraging, discouraging, and neutral expressions on event-related brain electrical activity during a visual selective attention task in which targets were determined by location, shape, and color. Although the expressions preceded the attentional task, both encouraging and discouraging messages elicited a similar long-lasting brain emotional response present during the visuospatial task. In addition, encouraging expressions were able to alter the customary working pattern of the visual attention system for shape selection in the attended location, increasing the P1 and the SP modulations while simultaneously fading away the SN.

**Conclusions/Significance:**

This was interpreted as an enhancement of the attentional processes for shape in the attended location after an encouraging expression. It can be stated, therefore, that encouraging expressions, as those used in sport practice, as well as in many other contexts and situations, do seem to be efficient in exerting emotional reactions and measurable effects on cognition.

## Introduction

The interplay between emotion and cognition has preoccupied philosophers and classical writers for many centuries. Even though, it is only recently that it has become the subject of scientific inquiry, investigations related to this question having been growing up in the past 20 years. Unfortunately, however, there is still a lack of theoretical and methodological background for this matter, so that any one study can provide, at best, only a very partial answer to the extremely complex relation between affect and reason [1].

The concept of emotion presents many puzzles, beginning with James's [2] still unanswered question what is an emotion. It is also puzzling the array of vastly different theories about the nature of emotion that have arisen. However, a distinction should be made between emotions and moods or ‘feelings’ [3]. Feelings or moods are coherent conscious states considered as a ‘long-term response’ in comparison to emotions, which are rapid and more automatic responses to an emotional stimulus at a low level of consciousness [4]. Emotions are typically more intensive than moods [5], the latter typically having a lack of the pronounced facial expression and changes in autonomic activity associated with emotions.

The majority of the literature on the affection-cognition interplay has focused on the effect of abnormal mood, such as clinical depression and anxiety disorders [6]. Nonetheless, an increasing number of scientists believe that feelings and emotions have to be studied in healthy subjects because feelings and emotions are always at hand and represent a pivotal point to understand the role of emotions in human cognition and behavior [e.g. 6], [7]. As feelings and emotions permeate people's daily lives, cognitive processes are bound to be performed in the presence of some affective or emotional state [8]. Hence, this is a main reason for studying how cognition is affected by affection.

Several studies have been interested in how emotional states affect different cognitive functions. In this regard, some initial data suggested that negative states might improve executive functions [9]. However, there is a bigger deal of evidence that cognitive processing is better enhanced by positive mood [10]–[13]. These studies have found that positive moods improve performance in a wide range of tasks, particularly those where cognitive flexibility is required [13], [14], [15], enhancing strategic retrievals, initiation of actions, inhibition of previous dominant responses and the ability to switch search strategies [6], [16].

There is a wide range of different procedures to induce an emotional state. The most common have been to have participants watch a film [17], [18], read funny cartoons, or experience success on an ambiguous task [8]. Other studies used participants' self-referent statements [19], music [20]–[23], faces expressing emotions [16], [24], or asked participants to recall an autobiographical memory with particular emotional connotations [e.g. 13].

On the other hand, language is a commonplace in people's every day events, playing a critical role in human relationships [25]. Within our daily lives, we can probably recall several moments in which a quick chat or a message changed our mood. A very common and well-known situation is the use of linguistic expressions to encourage (or discourage) performance in competitive contexts, such as sport practice. It is also not rare the use of self-encouragement messages in daily life by means of inner speech. Encouraging expressions are also frequent in politics (e.g., “*Yes, we can*”), as well as in many other contexts. Therefore, language is thought to be a suitable and powered factor not only to induce emotional states, but also to subsequently affect performance or cognitive processing. Surprisingly, however, there is a lack of studies on the effects of emotional states induced by language on cognitive processing. To the best of our knowledge, only one study has investigated the effects of the emotional state induced by language on cognition. Brosch and colleagues [26] studied the effect of anger prosody in visual attention. These authors reported cross-modal modulation of visual attention by auditory affective prosody, reflected in a reduction of time reaction response for stimuli presented in the right visual hemifield. Even though, the auditory material of this experiment consisted of pseudowords pronounced with either anger or neutral prosody, instead of real words or meaningful expressions. In addition, no positive emotional effects were studied.

The main aim of the present study is to fill this gap by investigating whether an emotional state can be induced by linguistic expressions and may affect cognitive processing. To study a specific cognitive function, we focused on visual selective attention. Very recently, several studies have reported that positive states are able to affect attention, increasing the breath of the attentional focus in a flanker paradigm [22], [24], reducing the “attentional blink” [27], and even overcoming the loss of awareness in patients with visual neglect [23]. Accordingly, visual selective attention appears vulnerable to emotional states. Furthermore, as is the case for encouraging (or discouraging) expressions, visual selective attention would also be commonly present in sport practice (at least in a number of sports).

A suitable methodological approach for these purposes is the recording of event-related brain potentials (ERPs). ERPs permit on-line measurements of electrical brain activity as visual attention processing unfolds over time. Certainly, much has been learnt about selective attention, particularly in the visual domain, with the ERP technique [e.g. 28], [29], [30], constituting a valuable tool in the measuring of very specific and subtle cognitive processes related to visual attention. In this regard, the ERP literature has provided clear evidences of the modulation of certain ERP responses to attended features of the stimuli. Namely, visual spatial attention boosts the P1 and/or N1 components amplitude, whereas attention to non-spatial features, such as shape or color, usually relates to processes occurring later in time, as a selection negativity (SN) and/or a selection positivity (SP) [29]. A hierarchical organization usually occurs, in the sense that non-spatial features modulate attention only in the attended location [31].

Data from the present study would help to better understand the emotion-cognition interplay in relatively frequent situations. This is the case of receiving linguistic expressions aimed at inducing emotional states that, in turn, would influence cognition and, hence, performance. The present study will aid at determining if linguistic expressions of either type (positive or negative) do actually exert objective and measurable effects on cognition, as actually intended, or, to the contrary, these expressions are rather vain or useless.

## Results

### Behavioral data

Reaction times (RTs) to target stimuli presented after positive expressions (mean: 606.09 milliseconds (ms), Sd: 59.96) were very similar to RTs to stimuli presented after neutral expressions (mean: 606.71 ms., Sd: 56.45). The RTs to stimuli presented after negative expressions were slightly shorter (mean: 597.68 ms, Sd: 48.67). The average percentages of omissions were relatively high for all the stimuli. For stimuli presented after negative expressions it was a little lower (25.99%) than for stimuli presented after positive expressions (27.87%) or after neutral expressions (28.28%). The average percentage of false alarms (FAs) was fairly small, with the smaller rate for neutral condition (6.6%), followed by positive condition (8.2%) and negative condition (9.3%). However, although there seemed to be a conservative strategy together with a better performance after negative expressions, no significant differences were observed between conditions either in RTs or in error rates (average percentage of omissions and FAs) as a function of the type of preceding expressions (*F*s_(1,46)_ between 1.26 and 1.86, *p* always >.1).

### ERP data

#### Visual selective attention effects

In line with a number of previous reports [e.g., 29]; [31], we found an overall effect of location, and of shape and color attention in the attended location. Our results are therefore supporting a hierarchical organization of visual selective attention, evidenced both by differences in the timing of the selection processes based upon spatial versus non-spatial features, as much as by the absence of non-spatial attention effects in the unattended locations. Overall, the early selection based on the location enhanced the P1 component, whereas attention to color and shape mainly induced a SN and a SP only for stimuli in the attended location. These results were supported by significant effects of the electrode by location interaction in the 120–144 and 144–168 ms windows (*F*s*_(26,598)_* = 5.37 and 7.14, respectively, *p*s<.05), as much as by significant effects in the 216–240 ms window of the interactions electrode by location by shape (*F_(26,598)_* = 4.11, *p*<.01) and electrode by location by color (*F_(26,598)_* = 3.96, *p*<.05). As these effects are in line with those extensively reported in previous literature, they will not be further detailed here. In the following, only results in which emotion yielded significant effects, either alone or in interaction, will be described.

#### Overall emotional effects

**Figure 1:**
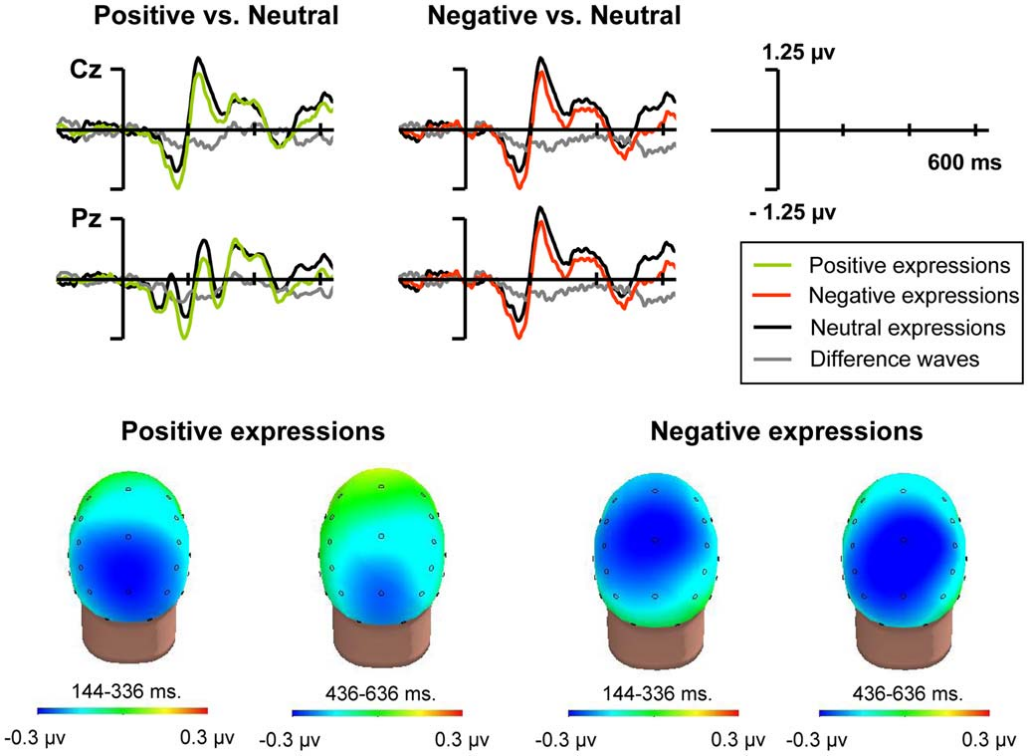
Overall effects of the expressions on subsequent brain activity. ERPs to stimuli of the visuospatial task as a function of the type of preceding expression. Overall effects of the expressions, not interacting with visual selective attention. *Top*. ERP waveforms at a selection of electrodes. *Bottom*. Difference maps of the effects in the 144–336 and 436–636 time windows.

Significant effects of type of expression, either alone or interacting with electrode, on the ERP fluctuations time-locked to visual stimuli appeared for the periods 144–336 ms and 436–636 ms (all *F*s*_(2,46)_* for type of expression alone between 2.43 to 6.37, *p*s always <.05; all *F*s*_(52,1196)_* when interacting with electrode between 2.18 to 3.61, *p*s between <.05 and <.01), revealing overall emotional effects. Figure 1 outlines these effects on the ERP fluctuations to visual stimuli as a function of the type of preceding expression, regardless of the attended features of the stimuli. As it can be seen, there is an emotional long-lasting negative effect along the whole epoch, starting around 130 ms and displaying a mainly central distribution, albeit the effects of positive expressions appeared slightly more posterior. Along the 336–436 segment, only a trend for significance could be found for the factor type of expression (*F*s*_(2,46)_* 3.42, *p* = .08). Although this is probably caused by noise, or by alternative random factors, we decided to in-depth analyse these fluctuations by considering two windows, based on significant results only: 144–336 and 436–636 ms.

Post-hoc pair-wise ANOVAs of these effects on these new widows revealed that there were no differences between the negative and the positive conditions (*F*s_(1,23)_ 0.05 and 0.13 for the early and the late window, respectively, *p*s always >.1), whereas both the positive and the negative conditions differed significantly from the neutral condition (*F*s_(1,23)_ between 6.54 and 8.63, *p*s always <.05, Bonferroni corrected). When the two time windows were compared both within the positive and within the negative conditions, separately, no significant differences were found (*F*_(1,23)_ = 0.8 and 0.53, for the positive and the negative conditions, respectively, *p*s always >.1), further supporting the above assertion that the activity in the two time periods reflects the same phenomenon, which could therefore be described as a long-lasting negativity starting at about 130 ms and covering the rest of the epoch. Despite subtle differences in topography between conditions, statistical analyses clearly support that this fluctuation was mostly common to visual stimuli occurring after both positive and negative expressions.

**Figure 2:**
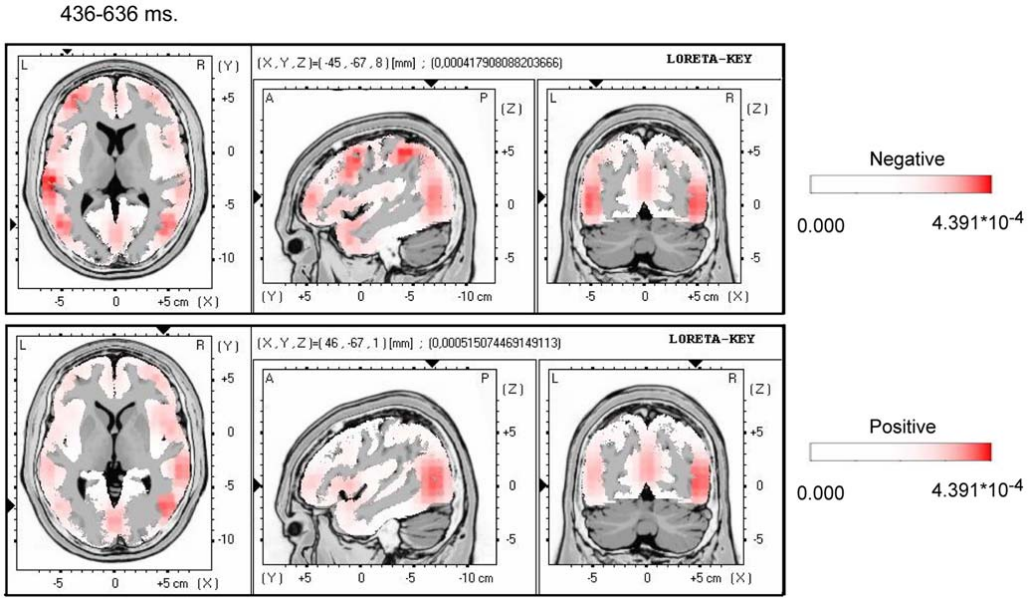
Three-dimensional location of the overall effects of the expressions. Three-dimensional location of the regions proposed by LORETA (low-resolution brain electromagnetic tomography [32]) as possible sources of the effects observed in Figure 1.

The next step was to three-dimensionally locate the cortical sources of these overall emotional effects in order to determinate their possible origin. To that aim, the *low-resolution brain electromagnetic tomography* (LORETA) was applied to ERP difference waves. LORETA is a three-dimensional, discrete linear solution for the EEG inverse problem [32]; solutions are projected on the Talairach standard brain [33]. The 436–636 window was selected for these analyses in both the positive and the negative conditions. As can be seen in Figure 2, with slight differences, both types of emotional expressions yielded a highly similar pattern of results, the solutions suggesting the medial temporal regions as the main origin of the overall emotional effects, even if other regions such as the fronto-polar portions might also be involved. A bilateral implication can be assumed in both conditions; apparent differences in lateralization in the figure should be discarded based on the statistical comparisons between these two conditions reported above.

#### Effects of affective expressions on visual selective attention

**Figure 3:**
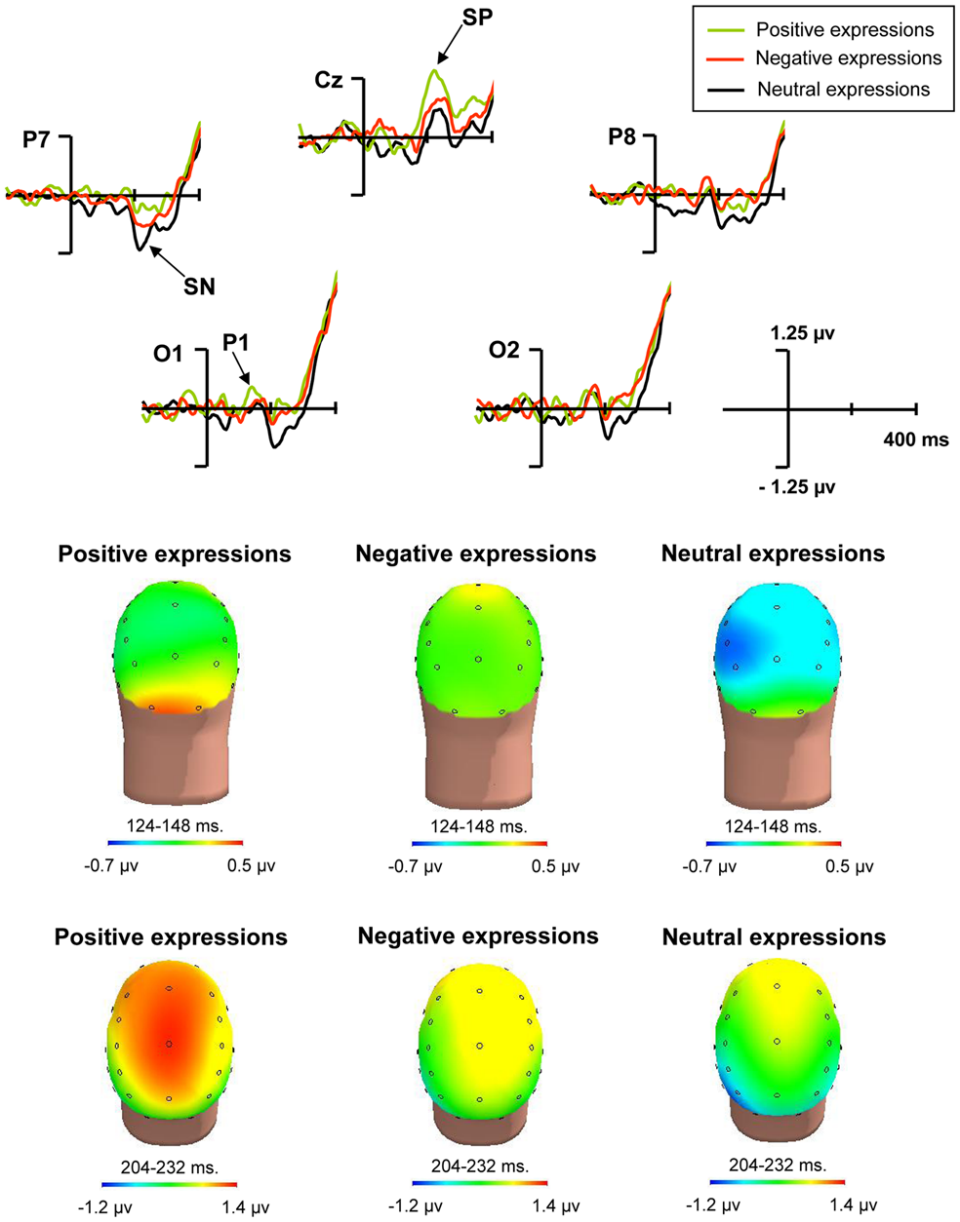
Effects of the expressions interacting with visual selective attention. ERPs to stimuli of the visuospatial task as a function of the type of preceding expression. Effects of the expressions interacting with visual selective attention. *Top*. ERP difference waveforms at a selection of electrodes. Difference waves were computed subtracting the waveforms to attended minus unattended shape, always in the attended location. *Bottom*. Difference maps of these effects in the 124–148 (P1) and 204–232 (SN and SP) time windows.

The overall analyses revealed significant interaction of type of expression, location, shape, and electrode in the time windows from 120 to 192 and from 216 to 288 ms, comprising the P1, SN and SP components (*F*s*_(2,46)_* between 3.51 and 7.19, *p*s between <.05 and <.01). No other significant effects for the interactions between type of expression and attentional features were observed. As can be seen in Figure 3, the P1 component was sensitive to experimental manipulations. Comparing conditions, an enhancement of the P1 component was observed to the attended shape in the attended location after the positive expressions. This was supported by significant effects of the type of expression by electrode by location by shape (*F*_(2,46)_ = 1.8, *p*<.05) in the time window established for the P1 (124–148 ms). Even though, post-hoc statistical analyses did not support a difference between types of expressions at the O1 electrode, where this effect was more apparent (*F*_(1,23)_ = between 1.03 and 2.45, *p* always >.1).

In the time window established for the SN and SP components (204–232 ms) a significant interaction was also found between type of expression, location, and shape (*F*_(2,46)_ = 7.66, *p*<.001). A lack of the SN component in the positive condition can be observed, together with an apparent SN component in both the neutral and the negative conditions. This effect appeared especially patent at the P7 electrode. Post-hoc pair-wise comparisons at this electrode revealed significant differences between positive and neutral conditions (*F*_(1,23)_ = 11.38, *p*<.05, Bonferroni corrected). The negative condition, however, did not differ significantly either when compared to the neutral condition (*F*_(1,23)_ = 1.83, *p*>.1), nor relative to the positive one, even if in the later case a trend for significance was found (*F*_(1,23)_ = 3.31, *p* = .08), which nevertheless does not hold after Bonferroni correction. On the other hand, it was evident an enhancement of the SP component in the positive condition, particularly at the Fz and Cz electrodes. Using the latter for post-hoc pair-wise comparisons, they were found significant differences between positive and neutral conditions (*F*_(1,23)_ = 10.12, *p*<.05, Bonferroni corrected), whereas there were no significant differences either between negative and neutral conditions (*F*_(1,23)_ = 1.23, *p*>.1), nor between negative and positive conditions (*F*_(1,23)_ = 2.51, *p*>.1). Overall, main effects of affective expressions on the P1, the SN, and the SP components are supported. Although post-hoc analyses did not substantiate a difference between conditions, the P1 to the attended shape in the attended location appeared larger after positive expressions. Thereafter, the SN seems to fade away whereas the SP increases noticeably to the attended shape in the attended location after positive expressions. The effects of negative expressions could be roughly described as resembling to some extent those of the neutral condition for the P1 and SP components, together with a reduction of the SN, although statistical analyses would support a rather intermediate position between the neutral and the positive conditions.

## Discussion

The main aim of the present study was to investigate whether the ERP components related to selective attention can be modulated by preceding positive, neutral, or negative linguistic expressions. Two main results can be reported. On one hand, we found an overall long-lasting brain response common to both positive and negative linguistic expressions, but not interacting with attentional features of the stimuli. On the other hand, data showed that positive expressions were able to modulate differentially the neural mechanisms of visual selective attention, particularly of attention to shape in the attended position.

It appears therefore that either type of emotional linguistic expression presented in this experiment was able to modulate participants' brain activity, yielding a long-lasting central negativity. Apparently, both positive and negative expressions were powerful enough to activate several brain areas, mainly of the medial temporal regions. This activation might involve, therefore, portions of the insula as well as other structures implicated in emotional information processing [e.g. 3], [34], [35], [36], favouring the emotional nature of this response. Moreover, this modulation has been found to occur for brain activity time-locked to subsequent events occurring a time after the occurrence of the linguistic expressions, proving consequently the long-term effectiveness of these expressions. The fact that the effects of both positive and negative expressions could not be disentangled at this level suggests that this is an overall emotional response, common to either type of expression with emotional valence. Indeed, the insula, particularly in its middle part, has been associated with emotional responses as much as visceral and somatic responses accessible to consciousness and elicited by emotional stimuli [37], [38]. The fact that insular activations have been shown for such contrasting emotions as romantic love and hate [39], [40] would be in line with our findings that both positive and negative expressions elicited this response.

Our first finding would be, therefore, that linguistic expressions similar to those used in certain situations, such as competitive contexts (among many others), are capable of eliciting a long-term emotional reaction. This would be true even if the subject is aware of the possible lack of connection between the emotional expressions and their real performance, again resembling what could occur in contexts where these expressions are commonly used. In our experiment, the instructions never established a relationship between the expressions and actual execution, the former being rather treated as constituting the material of a different task to be performed in addition to the visual attention task. This finding supports, accordingly, the power of language to generate emotional states. This overall emotional reaction, however, did not interact with visual selective attention processes.

Other effects, specific for the type of emotion of the expressions, interacted with selective attention. Following positive expressions, the P1 component amplitude to the attended shape in the attended location appeared larger, as compared to the negative and neutral conditions. At the same time, a subsequent SN component to this feature seemed to evade in the positive condition, being it apparent to some extent in the negative condition and, noticeably, in the neutral condition. A patent increase of the SP also occurred in the positive condition in comparison to the other two conditions. Negative expressions, on the other hand, induced a more ambiguous pattern of results, with no observable effects in the P1 component, a salient reduction of the SN and no effects on the SP. Accordingly, and in agreement with previous literature (reviewed in the introduction), the most reliable and prominent effects of emotion on cognition appeared to be linked to positive emotions.

Although the enhancement of the P1 component after positive expressions was not significant in post-hoc analyses, the fading of the SN component and the SP component boosting, which were significant, could be consistent with an actual P1 enhancement, all these data conforming a coherent pattern. This could be described, even if somehow speculatively, as a specific improvement or enhancement of the attentional processes for shape selection in the attended location when preceded by a positive linguistic expression. In this regard, the lack of an SN, together with a P1 enhancement, might be suggestive of shape selection taking place, at least partially, in the dorsal visual stream when preceded by positive expressions, therefore occurring earlier and at variance with the customary implication of the ventral stream to process this non-spatial visual feature [e.g. 29], [31]. This striking result, however, would not be totally unprecedented. In fact, the processing of shape attributes by the visual dorsal stream has occasionally been reported to occur under particular circumstances (e.g. when a threatening stimulus moves [41]). Further, this atypical pattern has never been reported previously for color selection. In line with this, no effects of this kind were observed here for color. The SP enhancement, on the other hand, would also be in consonance with an improvement of the attentional processes for shape in the attended location after an encouraging expression.

Several authors have proposed that positive emotional states are related with the dopamine system activation and its projections from the ventral tegmental area (VTA) to the prefrontal cortex [e.g. 6], [42], [43]. At the same time, high dopamine levels in specific portions of the prefrontal cortex have been related with the effect of positive feelings in cognitive functions [8], [16], [44], [45]. Therefore, the dopamine system could be mediating our findings. Current theories on brain organization suggest that cognitive functions such as attention or language are organized in widespread, segregated but simultaneously overlapping networks [46], [47]. Specialized sectors of the prefrontal cortex have a distinct set of connections and complementary roles in cognition, memory an emotion. The effect currently found on visual selective attention could be related to phenomena primarily occurring at frontal structures, where networks devoted to both visual attention and emotional processing may overlap. Interestingly, moreover, it has been reported that emotional regions in the prefrontal cortex modulate the activity of parietal areas, enhancing the activation of attention-related parietal systems [23], [48]. Our results would support that these effects may be extended also to occipital regions, where several of our effects would be taking place [30].

As mentioned, color selection did not exhibit any modulation by linguistic expressions, as was also the case for location, even though the attended location was a prerequisite for the effects of expressions on shape selection to show up. On the other hand, following standard procedures in selective attention research, the responses by our subjects were only to target stimuli, which were defined by location, shape, and color, concurrently. This would be the reason why the effects of positive expressions on shape selection were not apparent in our subjects' performance. However, the bulk of the literature demonstrating that the P1, SN and SP modulations are related to specific visual selective attentional processes strongly supports our interpretation that shape selection is affected by encouraging expressions. It is expected, accordingly, that tasks predominantly based on shape selection will noticeably benefit from encouraging expressions.

It can be concluded, therefore, that linguistic expressions can elicit long-term emotional responses by themselves, i.e., even if they are not overtly related to one's own performance. Although both negative and positive expressions can evoke an overall unspecific emotional response, only the latter exhibited specific and noticeable effects on a subsequent visual selective attention task. Namely, encouraging expressions were capable of altering the customary working pattern of the visual system, apparently leading the dorsal stream to process a feature that is normally processed in the ventral stream, as is the case of shape. Overall, it can be stated that linguistic encouraging expressions, as those used in sport practice and in many other contexts and situations, do seem to be efficient in exerting emotional reactions and measurable effects on cognition.

## Materials and Methods

### Ethics Statement

The study was performed in accordance with the Declaration of Helsinki, and approved by the ethic committee of the Center for Human Evolution and Behavior, UCM-ISCIII, Madrid, Spain. Participants gave their written informed consent prior to the study and received reimbursal thereafter.

### Participants

Twenty-four subjects (16 women) ranging in age from 18 to 27 years (Mean = 19,9 years) participated in the study. All the subjects had normal or corrected-to-normal vision and did not report any neurological disease. Eighteen subjects were right-handed, with average handedness scores [49] of +77, ranging from +37 to +100, whereas 6 were left-handed, with average handedness scores of −57, ranging from −22 to −95.

### Stimuli

Experimental material consisted in 240 verbal expressions and a set of visual stimuli. The expressions contained between one and five words and were presented orally. They could contain neutral information or information aimed at inducing positive or negative emotions in the participants.

Verbal expressions were constructed as follows. First, an initial pool of 300 emotional and neutral expressions (1–5 words) was made up. A hundred of the expressions were encouraging expressions, giving information about positive personal characteristics, positive expressions (e.g. “You are the best”, “Go ahead!”). One hundred referred to neutral features of the task situation, neutral expressions (e.g. “Look at the screen”). The third hundred of the expressions were discouraging expressions, giving information about negative personal characteristics, negative expressions (e.g. “You are going to fail”, “You are a loser”). Twenty participants different from the participants in the ERP experiment but having similar socio-demographic characteristics scored the expressions in a 5-points scale, from “very negative” (1) to “very positive” (7). Finally, 240 expressions (80 positive, 80 neutral and 80 negative) were selected from the ratings provided by this group.

After expressions' selection, a female speaker recorded them using the Cool Edit Pro® program. The speaker was instructed to emit the expressions in natural speech, caring for appropriate prosody envelope for positive and negative expressions, respectively, as well as neutral prosody for neutral expressions. This was judged as achieved by all the authors in separate individual judgement sessions. All of the expressions had durations between 1 and 3 seconds (s) and were displayed using loudspeakers placed 65 centimetres (cm) from the subject. Within-session intensity levels of the linguistic expressions were adjusted individually, so that each participant could hear them clearly and comfortably.

Visual stimuli consisted of triangles and circles that could be either red or blue. Stimuli could be presented 5 cm to the right or to the left of a central fixation white cross. Each visual stimulus had duration of 100 ms, and was presented randomly in a trial-to-trial basis with an interstimulus interval (ISI) that varied at random between 150 and 600 ms (rectangular distribution). The 2400 visual stimuli were presented white-on-black on a computer monitor, controlled by Presentation® software. Subjects' eyes were 65 cm from the monitor. At that distance, the visual angle of the triangles was 1,1°×1,1°, and circles were 0,9° in diameter (equivalent area for both triangles and circles).

### Procedure

**Figure 4:**
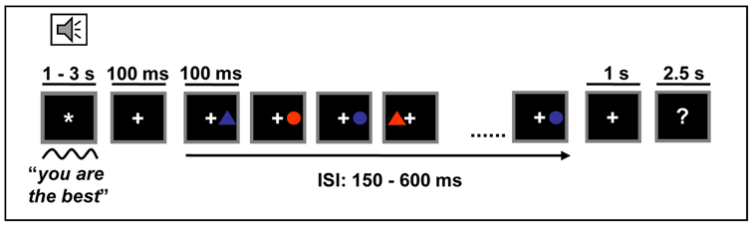
Stimulation procedures. Two tasks were to be done: First, hear an expression (encouraging, discouraging, or neutral) and keep it in mind, since it could be required to be rehearsed after the end of the visuospatial task. A short (10 trials) visuospatial task then followed, and a button had to be pressed as soon as a target (defined by location, shape, and color) appeared in the screen.

As sketched in Figure 4, the stimulation procedure was as follows. Each verbal expression preceded a short visuospatial task. During the presentation of the expressions, an asterisk appeared in the center of the screen as fixation point. Immediately after the end of an expression, the asterisk was substituted by a cross for 100 ms, thereafter starting a visuospatial task while keeping the fixation cross. Each short visuospatial task contained 10 trials, in which the occurrence of a target stimulus displayed a probability of 10% The target stimulus to be used for all the short visuospatial tasks was selected for each subject (counterbalanced), based on the combination of the visual features color, shape, and location. In the remaining, non-target stimuli, these features were settled randomly for each stimulus, with 50% probability for each of the two possible colours (red/blue), shapes (triangle/circle) and locations (left/right). After the last visual stimulus, the fixation-cross remained for 1 s. In 25% of the occasions, a central question mark appeared 1 s after the last visual stimulus, for 2.5 s. Subjects were seated in a comfortable chair and were instructed to listen the verbal expressions carefully and to maintain fixation on the central cross while subsequently discriminating the targets from the other visual stimuli. Subjects were told to press a button with the index finger as soon as the target appeared on the screen. The response hand was counterbalanced across subjects. In addition, they were told to repeat loudly the last verbal expression heard when a question mark appeared. No explicit instruction was given relative to any possible relationship between the expressions and subject's actual performance.

The experiment was divided in 3 blocks. Each block contained 80 verbal expressions, each followed by a short visuospatial task (800 visual stimuli per block). Within each block, linguistic expressions of either type (neutral, positive or negative) appeared in a random order, and none expression could appear twice for a given subject, so that each subject heard the whole array of 240 expressions in the experimental session.

### Electrophysiological Recordings

The electroencephalogram (EEG) was recorded from 27 tin electrodes mounted in an electrode cap (ElectroCap International®). The electrode sites were Fp1, Fp2, F7, F3, Fz, F4, F8, FC3, FC4, T7, C3, Cz, C4, T8, TP7, CP3, CP4, TP8, P7, P3, Pz, P4, P8, O1, O2, FT7 and FT8 of the revised 10/20 International System [50]. All electrodes were referenced online to the right mastoid and re-referenced offline to the average of the left and right mastoids. Bipolar horizontal and vertical electrooculograms (EOG) were recorded for artefact rejection purposes. Electrode impedances were always kept under 3 kilo-Ohms. The signal was recorded continuously with a band pass from 0.01 to 40 hertz.

### Data Analysis

Behavioral performance was measured in terms of reaction times (RTs) and error rates (omissions and false alarms –FAs-).

The continuous recording was divided into 1000-ms epochs starting 200 ms before each visual stimulus. Ocular correction for blink and eye movement was performed using the method described by Gratton et al. [51]. Artefacts rejection was performed, first, by semi-automatically eliminating the epochs over a range of ±100 microvolts, and by a visual inspection rejecting those epochs that still presented artefacts. Overall, the mean rate of rejected segments was 12%.

Separate averages were computed for each attended stimulus feature and for the three types of expressions (positive, neutral, and negative) separately. Based on preceding studies [e.g., 31], for a first over-all data exploration of the epoch, mean voltages of the ERPs to visual stimuli were calculated for consecutive 24 ms-wide time windows, starting at 120 ms after stimulus onset and continuing up to 336 ms. From 336 to 636 ms, time windows were 100-ms wide. Even though, given that the P1, SN, and SP components resulted of special interest, and their peak latencies were located midway between two time windows, two additional time windows were included in the follow-up analyses. For the P1 component the time window was established from 124 to 148 ms after stimulus onset, whereas for the SN and SP components it was from 204 to 232 ms (same window). Also as a consequence of the results of the analyses, two other additional windows were used, to better explore long-lasting emotional effects (144–336 and 436–636 ms; see above).

To study the mean voltage differences between conditions, analyses of variance (ANOVA) were computed, using the Greenhouse-Geisser epsilon correction for nonsphericity of the variance-covariance matrix. An ANOVA with five-repeated measures factors (type of expression, location, shape, color, and electrode) was first computed for all the time windows.

In order to three-dimensionally locate the cortical regions that were sensitive to experimental manipulations, *low-resolution brain electromagnetic tomography* (LORETA) was applied [32].
